# The efficacy and toxicity profile of metronomic chemotherapy for metastatic breast cancer: A meta-analysis

**DOI:** 10.1371/journal.pone.0173693

**Published:** 2017-03-15

**Authors:** Yangyang Liu, Feifei Gu, Jinyan Liang, Xiaomeng Dai, Chao Wan, Xiaohua Hong, Kai Zhang, Li Liu

**Affiliations:** Cancer Center, Union Hospital, Tongji Medical College, Huazhong University of Science and Technology, Wuhan, China; West China Second Hospital, Sichuan University, CHINA

## Abstract

**Purpose:**

The current meta-analysis aimed to summarize the available evidence for the efficacy and serious adverse events (AEs) associated with use of metronomic chemotherapy (MCT) in patients with metastatic breast cancer (MBC).

**Method:**

Electronic databases (PubMed, EMBASE database, Web of Knowledge, and the Cochrane database) were systematically searched for articles related to the use of MCT in MBC patients. Eligible studies included clinical trials of MBC patients treated with MCT that presented sufficient data related to tumor response, progression-free survival (PFS), overall survival (OS), and grade 3/4 AEs. A meta-analysis was performed using a random effects model.

**Results:**

This meta-analysis consists of 22 clinical trials with 1360 patients. The pooled objective response rate and clinical benefit rate of MCT were 34.1% (95% CI 27.4–41.5) and 55.6% (95% CI 49.2–61.9), respectively. The overall 6-month PFS, 12-month OS, and 24-month OS rates were 56.8% (95% CI 48.3–64.9), 70.3% (95% CI 62.6–76.9), and 40.0% (95% CI 30.6–50.2), respectively. The pooled incidence of grade 3/4 AEs was 29.5% (95% CI 21.1–39.5). There was no statistically significant difference observed in any endpoint between subgroups defined by concomitant anti-cancer therapies or chemotherapy regimens. After excluding one controversial study, we observed a trend showing lower toxicity rates with the use of MCT alone compared to use of MCT with other anti-cancer therapies (P = 0.070).

**Conclusions:**

Metronomic chemotherapy may be effective for use in patients with metastatic breast cancer. MCT used alone is possibly equally effective and less toxic than combination therapies. Well-designed RCTs are needed to obtain more evidence.

## Introduction

Although treatment strategies have continuously evolved over the past several years, the survival rates of patients with metastatic breast cancer (MBC) remain dismal, with a mean survival time ranging from only 2 to 3 years [1]. Metronomic chemotherapy (MCT) not only provides therapeutic effects, but also has a favorable toxicity profile and is economically feasibility. The low toxicity profile of MCT renders a better quality of life for patients, especially for those with recurrent disease [2,3], compared to standard chemotherapy regimens.

MCT refers to daily or frequent low dose administration of conventional chemotherapy drugs. It was first proposed by Hanahan et al. and has been constantly developing since [4,5]. Identified as an anti-angiogenesis therapy, it was originally thought that MCT worked by targeting only endothelial cells [6–9]. More recently, however, other mechanisms of action (e.g., inhibiting cancer stem cells and activating the immune system) have been found [10,11]. Traditional chemotherapy, in which the maximum tolerated dose is used, often exerts serious, detrimental side-effects and frequently surrenders to therapeutic resistance. In contrast, MCT maintains favorable anti-cancer activity and requires the use of less costly chemotherapeutic agents [6,12]. All of the aforementioned characteristics of MCT make it an ideal and efficacious therapy for use in MBC patients.

MCT research has been most commonly conducted on patients with breast cancer [13]. The first MCT study was conducted by Colleoni et al. in 2002 and it included 63 MBC patients treated with low-dose oral methotrexate and cyclophosphamide. Findings from this study showed an overall objective response rate (ORR) of 19%, an overall clinical benefit rate (CBR) of 32%, and a low incidence of grade 3/4 adverse events (AEs) [7]. Another MCT study showed weekly paclitaxel dosing resulted in a higher complete response (CR) rate compared to a standard 3-week schedule [14]. A series of single-arm, phase II clinical trials involving the various types of chemotherapeutic agents used in MCT have been conducted [15]. However, the results of these research studies have been conflicting. Additionally, some patients in these studies had been given anti-angiogenic drugs, hormonal therapies, and/or anti-inflammatory agents in addition to MCT [16–18]. This raises questions about whether these combinations are appropriate and if they result in an increased therapeutic efficacy and/or increased toxicity.

We conducted a meta-analysis to summarize the available evidence for the efficacy and AEs associated with use of MCT (used alone and also as part of a combination regimen) in patients with MBC.

## Method

### Search strategy

The following databases were searched for relevant studies: PubMed, EMBASE, Web of Knowledge, and the Cochrane database (updated to November, 21 2016). The key words or corresponding Mesh terms used to search the databases were: “breast tumor” or “breast tumors” or “breast cancer” or “breast cancers” or “breast neoplasms [Mesh]”and “metronomic” and “chemotherapy” or “chemotherapies” or “drug therapy [Mesh]”. We also screened reference lists of recently published trials and reviews to avoid overlooking any relevant articles. All published papers were restricted to the English language. In cases where there was overlapping data (e.g., data derived from the same clinical trials and contained in two or more publications), the most complete and updated report was selected for inclusion in this meta-analysis.

### Trial selection

Studies were screened independently by two authors (YYL and FFG). The inclusion criteria used to select studies included in this meta-analysis were: (1) phase II or III prospective clinical trials of MCT in patients with MBC, (2) average patient age greater than 18 years, (3) patients with normal hepatic, renal, and marrow functions, and (4) sufficient data provided about tumor response, progression-free survival (PFS), overall survival (OS) and adverse events (AEs). Clinical trials that combined MCT with other drug therapies were also included.

### Data extraction

The two investigators (YYL and FFG) independently extracted pertinent data, including tumor response, 6-month PFS (PFS-6) rate, 12-month OS (OS-12) rate, 24-month OS (OS-24) rate, and grade 3/4 AEs. Divergences were resolved by censure. Tumor response was assessed using the Response Evaluation Criteria in Solid Tumors (RECIST) criteria [19]. CBR reflects the proportion of patients with complete response (CR), partial response (PR), or prolonged stable disease (pSD) ≥24 weeks; ORR reflects those with CR or PR. Engauge Digitizer version 4.1 was used to ascertain survival data by digitizing figures if the information was not provided directly. AEs were evaluated according to the National Cancer Institute Common Toxicity Criteria (NCICTC). The incidence of AEs extracted from an individual study consisted of the sum of the different severe AEs that were recorded. Other information that was independently recorded included: first author’s name, year of publication, country, study design, registration number, age of subjects, MCT schedule, and number of assessable patients.

### Data analysis

We followed the Preferred Reporting Items for Systematic Reviews and Meta-analyses (PRISMA) checklist and guidelines for conducting this meta-analysis (S1 PRISMA Checklist). Summary measures of the above mentioned indicators have been presented in the form of incidence with the corresponding 95% confidence interval (CI). These measures were either directly extracted from the articles or calculated. Heterogeneity was tested by calculating Cochrane’s Q statistic and I^2^ statistic. When P≤0.10 or I^2^>50%, a random effects model was used to pool effect sizes of each study for heterogeneity. Otherwise, a fixed effects model was selected [20,21]. Sensitivity analysis was conducted by step-wise removal of single trials. Subgroup diversity was analyzed by using Q statistic. Visual inspection of funnel plot with Egger and Begg tests were adopted to assess publication bias [22,23]. Evidence quality for each endpoint was assessed by modified GRADE [24]. A two-tailed P-value of <0.05 was regarded as statistically significant. All the statistical analyses were performed using the Comprehensive Meta-Analysis program (Version 2, Biostat, Englewood, NJ, USA).

## Results

### Search results and study characteristics

**Fig 1:**
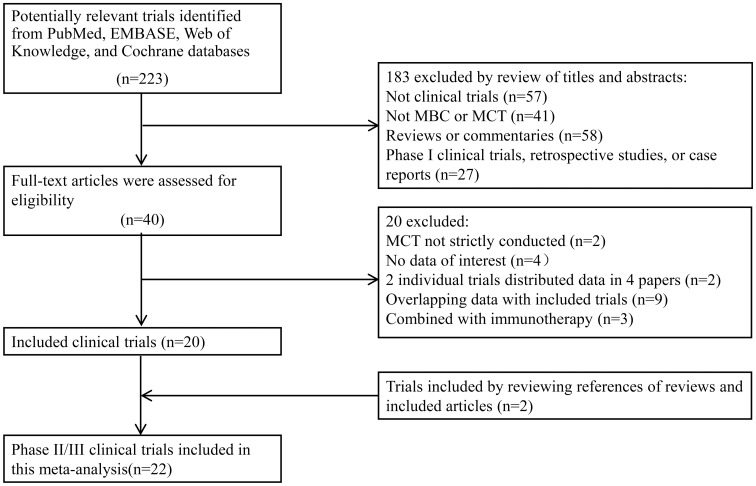
Flow diagram of the process used to select clinical trials. MBC-metastatic breast cancer; MCT-metronomic chemotherapy.

The flow diagram of the study is shown in Fig 1. A search in four electronic databases: PubMed, EMBASE, Web of Knowledge, and the Cochrane database, yielded 223 articles. By reading the titles and abstracts, 183 papers were excluded. As shown in Fig 1, the two most common reasons for study exclusion were lack of relevance to subject matter (i.e., not pertaining to MBC or MCT) and a study design that was not a clinical trial. Among the remaining 40 publications, 20 were excluded upon reading the full text. Reasons for exclusion were: 2 studies did not pertain solely to MCT; data of interest was not reported in 4 studies; the total data of 2 trials had been presented in 4 papers [25–28] (so all of the articles were included with trial number adjusted only); 9 papers presented overlapping data with other studies included in the meta-analysis; and 3 trials presented data from MCT combined with immunotherapy (the results of which could not be explained by the two newly-emerging anti-cancer methods [29–31]). Lastly, two additional studies that met the inclusion criteria were identified from the reference of articles [32,33].

Finally, 22 trials (24 publications), consisting of 4 randomized clinical trials and 18 single-arm clinical trials, with 1360 patients, were entered into our meta-analysis (Table 1). There were 12 trials in which MCT was used alone, 8 trials in which MCT was used in combination with other therapies (e.g., conventional anti-angiogenic drugs, hormonal therapies, or anti-inflammatory agents), and 2 trials in which patients were divided into two groups (one received MCT alone and the other group received MCT combined with other therapies). In summary, a total of 818 patients received MCT, 383 patients received MCT combined with other therapies, and 159 patients could not be grouped (see detailed MCT schedules and registration numbers in S1 Table). It should be noted that data about tumor response from 5 studies were omitted due to the lack of adherence to the RECIST criteria [3,7,25,28,34], so were AEs data for lacking adherence to the NCICTC guidelines [18,28,35–39]. The 2 trials in which patients were divided into two separate groups (MCT vs. MCT in combination with other therapy) did not present data separately for each individual group and therefore data from those studies were excluded from the subgroup analysis [25,28].

**Table 1:** Characteristics of the trials included in the meta-analysis.

Type^a^	Author, year	Country	Trial design	Schedule	Age (years) median (range)	Patients evaluated	Tumor response		PFS-6(%)	OS-12(%)	OS-24(%)	Grade3/4 AEs^e^	Evaluation criteria^f^
							OR	CB					
MCT	Colleoni 2002[7]	Italy	Single-arm phase II	CTX + MTX	57(36–80)	63	14	22	NA	NA	NA	12	WHO/ NCICTC
	Salem 2008[3]	Egypt	Single-arm phase II	CTX + MTX	56(37–72)	42	7	13	NA	NA	NA	8	WHO/ NCICTC
	Addeo 2010[34]	Italy	Single-arm phase II	VNB	75(70–84)	34	13	NA	NA	80.5	2.8	14	WHO/ NCICTC
	Stockler 2011[33]	Australia	Randomized phase II	Cap	62(NA)	107	21	NA	NA	NA	NA	32	RECIST/ NCICTC
	El-Arab 2012[36]	Egypt	Single-arm phase II	CTX + Cap	61(41–72)	60	13	NA	NA	58.7	13.8	NA	RECIST/ NA
	Fedele 2012[40]	Italy	Single-arm phase II	Cap	63(37–82)	58	14	36	NA	57.7	43.4	3	RECIST/ NCICTC
	Wang 2012[41]	China	Single-arm phase II	CTX + Cap	51(29–70)	66(68)^b^	20	35	40.9	65.1	31.8	18	RECIST/ NCICTC
	Yoshimoto 2012[35]	Japan	Single-arm phase II	CTX + Cap	61(32–82)	45(51)^b^	20	26	75.1	86.7	71.1	22	RECIST/ CTCAE
	De Iuliis 2015[42]	Italy	Single-arm phase II	VNB	76(69–83)	32	22	16	NA	NA	NA	0	RECIST/ NCICTC
	Martín 2015[32]	Spain	Randomized phase II	Cap	59(29–81)	97	31	56	NA	74.0	48.9	76	RECIST/ NCICTC
	Otsuka 2015[43]	Japan	Single-arm phase II	Irinotecan + TS-1	59(35–79)	40	16(34)^c^	NA	77.4	79.3	58.3	16	RECIST/ NCICTC
	Cazzaniga 2016[37]	Italy	Single-arm phase II	VNB + Cap	65(56–70)	80	22(74)^c^	39	52.5	NA	NA	NA	RECIST/ NA
Combination	Dellapasqua 2008[16]	Italy	Single-arm phase II	CTX + Cap Bevacizumab	58(35–75)	46	22	27(40)^c^	67.0(40)^c^	NA	NA	17	RECIST/ NCICTC
	García-Sáenz 2008[44]	Spain	Single-arm phase II	CTX + MTX Bevacizumab	49(30–71)	22	7	14	52.4	58.4	NA	5	RECIST/ NCICTC
	Wong 2010[45]	Canada	Single-arm phase I/II	CTX + MTX Deltaparin Prednisone	55(30–84)	41(40)^b^	7	10	22.2	50.0	27.4	13	RECIST/ NCICTC
	Licchetta 2010[38]	Italy	Single-arm phase II	CTX + megestrol acetate	72(45–86)	29	9	NA	NA	NA	NA	NA	RECIST/ CTCAE
	Montagna 2012[46]	Italy	Single-arm phase II	CTX + Cap Bevacizumab Erlotinib	47(32–64)	24(25)^b^	15	18	66.7	90.5	52.9	5	RECIST/ NCICTC
	Schwartzberg 2014[17]	America	Single-arm phase II	Fulvestrant + Cap	65(37–85)	41	10	24	73.0	87.0	68.3	18	RECIST/ NCICTC
	Perroud 2016[18]	Argentina	Single-arm phase II	CTX + Celecoxib	57(38–78)	20	1	11	30.0	25.0	10.0	8	RECIST/ CTCAE
	Rochlitz 2016[39]	Switzerland	Randomized phase III	CTX + Cap Bevacizumab	62(29–81)	74(68)^b^	37	NA	62.9	NA	NA	35	RECIST/ CTCAE
Either	Pectasides 2012 [27,28]	Greece	Single-arm phase II	Docetaxel (trastuzumab in HER2+)	61(27–87)	159	61	NA	62.3(122)^d^	75.4(122)^d^	49.2(122)^d^	NA	ECOG
	Colleoni 2006[25,26]	Italy	Randomized phase II	A:CTX + MTX	54(33–77)	86	18	36	48.2(112)^d^	75.9(112)^d^	34.8(112)^d^	23	WHO/ NCICTC
				B:CTX + MTX Thalidomide	55(31–78)	85	10	35				28	

MCT, metronomic chemotherapy; OR, objective response; CB, clinical benefit; PFS-6, 6-month progression-free survival; OS-12, 12-month overall survival; AEs, adverse events; NA, not available; CTX, cyclophosphamide; MTX, methotrexate; Cap, capecitabine; VNB, vinorelbine; TS-1, tegafur–gimeracil–oteracil potassium; HER, human epidermal growth factor receptor;

^a^Studies can be classified into metronomic chemotherapy group, combination group (combine MCT and other anti-cancer therapy) and either group(conducting either of them in different patients)

^b^Figure in round brackets represents the number of patients eligible for adverse events evaluation

^c^Figure in round brackets represents the number of patients eligible for corresponding clinical ending point

^d^Survival data were not grouped; figure in round brackets represents the total patients available for survival analysis

^e^The frequency are sum of different graded 3/4 AEs

^f^The evaluation criteria for tumor response and toxicity.

### Tumor response rate

**Fig 2:**
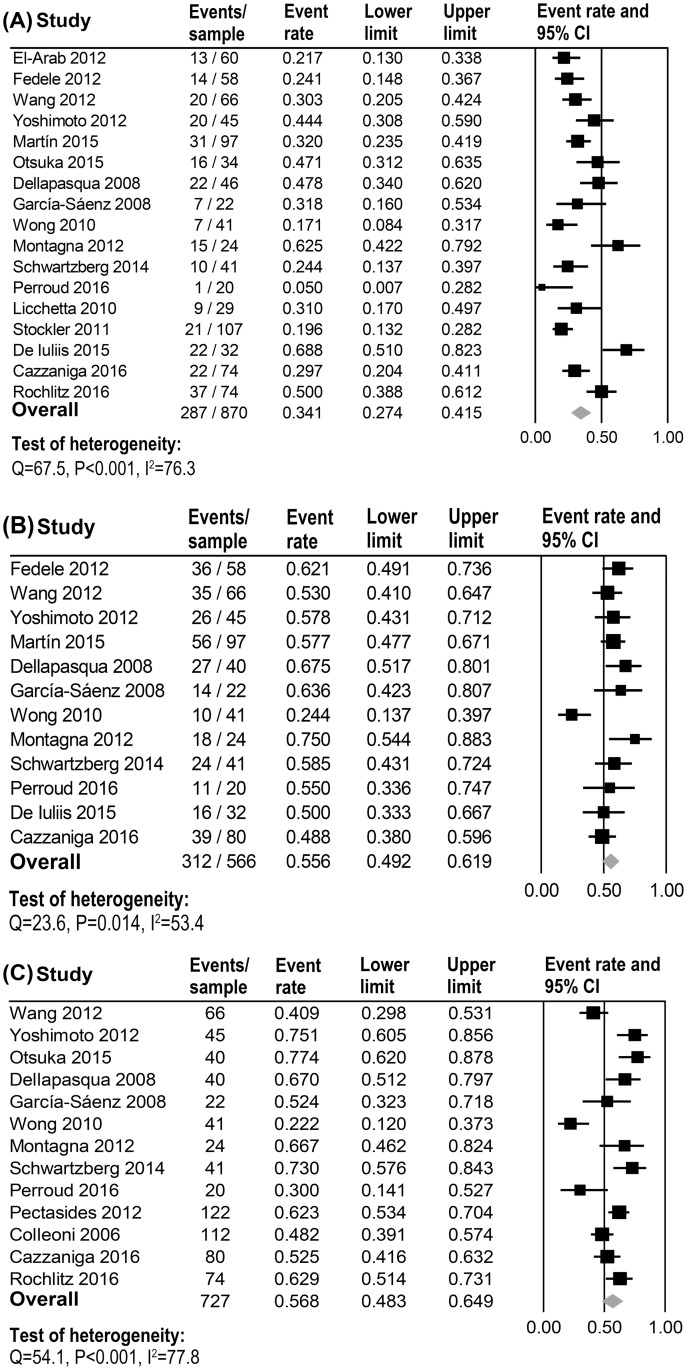
Objective response (A), clinical benefit (B) and 6-month PFS (C) of Metronomic Chemotherapy (MCT) for Metastatic Breast Cancer (MBC). Overall response: CR+PR; Clinical benefit: CR + PR + SD ≥24 weeks.

ORR data was extracted from 17 trials for this meta-analysis. The pooled ORR was 34.1% (95% CI 27.4–41.5) by using the random effects model (heterogeneity analysis: Q = 67.5, P<0.001, I^2^ = 76.3, Fig 2A). A subgroup analysis based on whether MCT was used alone or combined with other drug therapies. As shown in Table 2, there was no statistically significant difference in the ORR between MCT used alone and the combination schemes (33.5% vs. 34.2%, respectively, P = 0.925).

**Table 2:** Comparison of different clinical endpoints between the MCT and combination schemes.

	MCT assigned uniquely		Combination schemes		P value
	No. of trials	Incidence %(95% CI)	No. of trials	Incidence %(95% CI)	
OR	9	33.5(25.5–42.6)	8	34.2(23.2–47.3)	0.925
CB	6	55.0(49.9–60.0)	6	57.0(41.5–71.3)	0.807
PFS-6	4	61.6(43.8–76.8)	7	54.0(39.1–68.2)	0.513
OS-12	7	71.3(62.7–78.7)	5	65.2(39.4–84.4)	0.620
OS-24	7	38.1(24.0–54.5)	4	38.8(17.5–65.5)	0.963
Grade 3/4 AEs	10	27.2(16.1–42.2)	6	33.6(27.8–39.9)	0.418
Grade 3/4 AEs^a^	9	24.4(17.7–32.5)	6	33.6(27.8–39.9)	0.070

^a^Grade 3/4 AEs after removing a controversial trial.

The CBR was calculated using data from 12 clinical trials. The overall CBR was 55.6% (95% CI 49.2–61.9) as calculated by the random effects model (heterogeneity analysis: Q = 23.6, P = 0.014, I^2^ = 53.4, Fig 2B). Sensitivity analysis showed that most of the heterogeneity was derived from a trial conducted by Wong et al. [45] (heterogeneity analysis for the rest of the trials: Q = 9.1, P = 0.521, I^2^<0.001). There was no statistically significant difference in CBR of MCT used alone and in combination schemes (55.0% vs. 57.0%, respectively, P = 0.807); there was no statistically significant difference even when data from one controversial clinical trial was excluded from the analysis (MCT used alone vs. combined treatment: 55.0% vs. 63.6%, respectively, P = 0.075).

### Survival rate

Data for PFS-6 rate were available for analysis from 13 clinical trials. The overall PFS-6 rate was 56.8% (95% CI 48.3–64.9) as determined by the random effects model (heterogeneity analysis: Q = 54.1, P<0.001, I^2^ = 77.8, Fig 2C). There was no statistically significant difference in the PFS-6 rate between MCT alone and the combination schemes (61.6% vs. 54.0%, P = 0.513).

**Fig 3:**
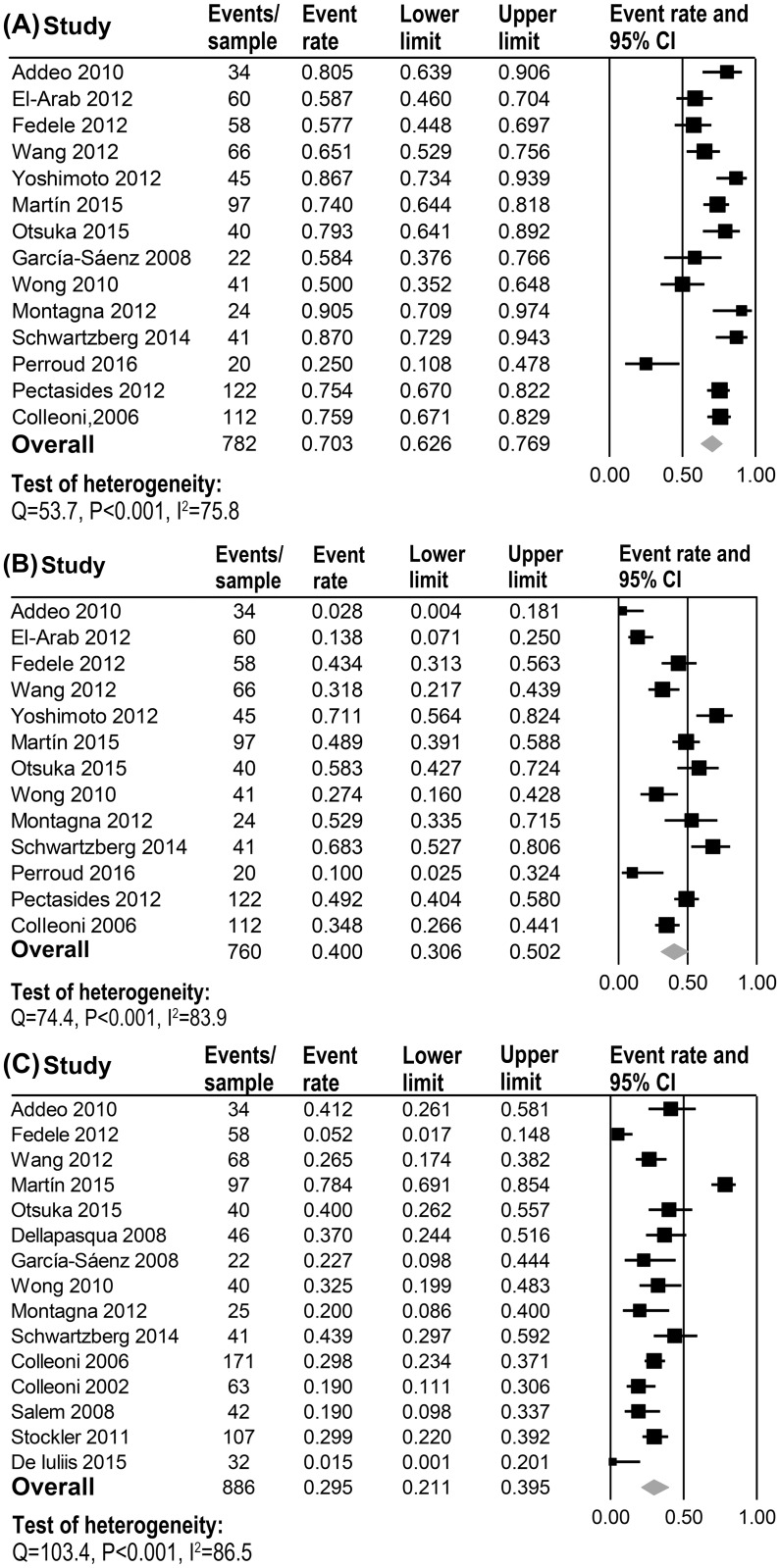
12-month OS (A), 24-month OS (B) and grade 3/4 side adverse events (C) of Metronomic Chemotherapy (MCT) for Metastatic Breast Cancer (MBC).

Data for calculation of the OS-12 rate were obtained from 14 trials. The pooled OS-12 rate was 70.3% (95% CI 62.6–76.9) with the random effects model (heterogeneity analysis: Q = 53.7, P<0.001, I^2^ = 75.8, Fig 3A). A statistically significant difference was not detected in the OS-12 rate between MCT alone and the combination schemes (71.3% vs. 65.2%, respectively, P = 0.620).

Data from 13 clinical trials showed the overall OS-24 rate was 40.0% (95% CI 30.6–50.2) by using the random effects model (heterogeneity analysis: Q = 74.4, P<0.001, I^2^ = 83.9, Fig 3B). There was no statistically significant difference in the OS-24 rate between MCT alone and the combination schemes (38.1% vs. 38.8%, respectively, P = 0.963).

### Grade 3/4 AEs rate

Data for grade 3/4 AEs were available from 15 trials. The pooled rate of grade 3/4 AEs was 29.5% (95% CI 21.1–39.5) as calculated by the random effects model (heterogeneity analysis: Q = 103.4, P<0.001, I^2^ = 86.5, Fig 3C). Subgroup analysis showed serious polarized heterogeneity for MCT (Q = 98.8, P<0.001, I^2^ = 90.9) and combination schemes (Q = 5.3, P = 0.379, I^2^ = 5.9). Sensitivity analysis demonstrated that a total or subgroup of heterogeneity could be attributed primarily to one clinical trial [32] compared to others. After removing the data from that trial, we observed a trend showing a lower AEs rate favoring MCT used alone as compared to the combined schemes (24.4% vs. 33.6%, respectively, P = 0.070).

### Subgroup analysis based on different chemotherapies

A subgroup analysis was performed among cyclophosphamide + methotrexate (CM), capecitabine, and other drug based regimens, but no statistically significant difference was found (Table 3).

**Table 3:** Comparison of different clinical endpoints among CM, Cap or other regimens based MCT schemes.

	Cap		CM		Other		P value
	No. of trials	Incidence % (95% CI)	No. of trials	Incidence % (95% CI)	No. of trials	Incidence % (95% CI)	
OR	11	0.337(0.267–0.416)	2	0.233(0.120–0.403)	4	0.385(0.181–0.639)	0.441
CB	8	0.579(0.529–0.628)	2	0.424(0.123–0.794)	2	0.519(0.385–0.651)	0.563
PFS-6	7	0.620(0.522–0.709)	3	0.403(0.239–0.592)	3	0.588(0.362–0.781)	0.133
OS-12	7	0.738(0.635–0.821)	3	0.629(0.436–0.788)	4	0.681(0.465–0.840)	0.539
OS-24	7	0.461(0.320–0.608)	2	0.329(0.259–0.408)	4	0.282(0.115–0.543)	0.237
Grade 3/4 AEs	7	0.324(0.173–0.523)	5	0.261(0.209–0.320)	3	0.311(0.138–0.559)	0.734
Grade 3/4 AEs^a^	6	0.268(0.180–0.378)	5	0.261(0.209–0.320)	3	0.311(0.138–0.559)	0.902

CM, methotrexate + cyclophosphamide; Cap, capecitabine

^a^Grade 3/4 AEs after removing a controversial trial.

### Sensitivity analysis

Sensitivity analysis did not demonstrate that overall effect sizes had been significantly altered by any clinical trial (S1 Fig), while there are two trials obviously contributing heterogeneity as mentioned above.

### Publication bias and quality assessment

There was no evidence of publication bias for the overall tumor response rate, survival rate, and grade 3/4 AEs rate (Table 4 and S2 Fig). Evidence quality for each clinical endpoint in this meta-analysis was graded to very low by using the modified GRADE (S2 Table).

**Table 4:** P value of Egger and Begg assessing publication bias.

	OR	CB	PFS-6	OS-12	OS-24	Grade3/4 AEs	Grade3/4 AEs^a^
begg	0.902	0.537	1.000	0.913	0.200	0.167	0.101
egger	0.790	0.702	0.848	0.781	0.236	0.113	0.062

^a^Grade 3/4 AEs after removing a controversial trial.

## Discussion

For over a decade, metronomic chemotherapy (MCT) has played a role in the anti-cancer arena. The findings from this meta-analysis of 22 clinical trials showed that the overall ORR, CBR, and PFS-6 rate for MBC patients treated with MCT were 34.1% (95% CI 27.4–41.5), 55.6% (95% CI 49.2–61.9), and 56.8% (95% CI 48.3–64.9), respectively. These rates were higher than those reported in another systematic review, in which the median ORR, CBR, and PFS were 26.0%, 46.5% and 4.6 months, respectively, summarized from various cancers [13]. The pooled grade 3/4 AEs rate of 29.5% (95% CI 21.1–39.5) seemed to be a little high; this may be attributed to our taking into account the different kinds of observed AEs. Similar to the findings of our study, results from a recently published meta-analysis also show a better toxicity profile with the use of a lower dosage of capecitabine [47]. Most of the of MBC patients in this study were either pretreated or had chemotherapy resistance; these findings plus the OS data are optimistic and are further supported by the results of a recent, randomized, phase II study (NCT0141771) aimed to prove that MCT was effective and less toxic in comparison to standard chemotherapy [48].

Subsequently, we compared the outcomes between patients treated with MCT alone to those who were treated with MCT and another anti-angiogenic, anti-hormonal, or anti-inflammatory agent. Much to our surprise, there was no statistically significant difference observed in any of the endpoints. This observation is consistent with the findings from a randomized controlled trial (RCT) brought into our meta-analysis, in which MBC patients accepted metronomic, low-dose oral cyclophosphamide and methotrexate plus or minus thalidomide [25]. It is unclear why the combination schemes worked well in preclinical studies but not in clinical studies. Outwardly, MCT combined with targeted therapy showed better clinical value, but the statistical significance is indefinite, as single-arm trials lack available control groups and further subgroup analysis cannot be easily conducted for limited studies here [16,39,44,46]. On the other hand, a study conducted by Saloustros et al. evaluating the salvageability of metronomic vinorelbine plus bevacizumab, was stopped prematurely due to minimal activity in terms of ORR (7.7%) [49]. Additionally, findings from a phase III RCT included in this analysis comparing bevacizumab-based MCT with bevacizumab-based standard chemotherapy showed pessimistic results: ORR (50% vs.58%, respectively, p = 0.45), median PFS (8.5 vs.10.3 months, respectively, p = 0.90), and serious AEs rate (24% vs. 25%, respectively, p = 0.96) [39]. Another RCT analyzing bevacizumab-based MCT versus pure standard chemotherapy in MBC patients also showed no significant variation for PFS and OS [50]. These studies indicate the combination schemes are meaningless to some degree.

Schwartzberg et al. selectively administered fulvestrant with MCT to hormone receptor-positive, HER2-negative MBC patients. Findings from his study showed a moderate tumor response rate and a relatively prolonged survival time (median PFS 14.98 months [95% CI 7.26-upper limit not estimated] and median OS 28.65 months [95% CI 23.95- upper limit not estimated], respectively) [17]. It is also worth noting the results of a study by Montagna et al. in which erlotinib was added to the regimen of patients who were potentially overexpressing epidermal growth factor receptor (EGFR), showing better therapeutic effects, ORR 62% (95% CI 41–81) and CBR 75% (95% CI 53–90) [46]. In addition, the future direction of MCT can be guided by the development process of a combination approach of hormonotherapy and standard chemotherapy, which has progressively become an accepted therapy following the implementation of new drugs and discovery of multiple blocking mechanisms [51,52].

The sensitivity analysis showed that only one trial [45] was found to contribute to most of the heterogeneity for CBR. However, that study is methodologically sound with regard to its study design, execution, and evaluation, hence we found no compelling reason to exclude that trial.

Another trial [32] was found to impact the heterogeneity in the incidence of grade 3/4 AEs; we re-examined that study. Large doses of capecitabine (800 mg/m^2^ twice daily) were administered as monotherapy to MBC patient; other studies rarely use such large doses for the MCT model. Also, in a similar RCT, a continuous capecitabine regimen with lower doses (650 mg/m^2^ twice daily) was well tolerated [33]. So there is a high risk of generating wrong result when including this research [32]. More importantly, after removing that study from this meta-analysis, we observed a trend showing a lower severe AEs rate in MCT given alone compared to MCT administered with other therapies (24.4% vs. 33.6%, respectively, P = 0.070). The small amount of heterogeneity (Q = 5.3, P = 0.379, I^2^ = 5.9) in the combination schemes also supports that severe AEs existed in these situations. Findings from a later RCT showed that toxicities associated with the combination scheme were as severe as toxicities from standard chemotherapy [39]. In the included RCT comparing MCT alone and combination schemes, although no statistically significant difference was observed for grade 3/4 AEs, there was a higher incidence of mild AEs for the latter [25]. The quality of life for MBC patients is of major importance so one should cautiously balance the contradiction between therapeutic effects and AEs when designing future studies.

Due to lack of consensus about drug selections and corresponding dosages, large RCTs are recommended. Munzone et al. summarized some ongoing clinical trials and the forthcoming results may be helpful [2,15]. Meanwhile, according to the principles of precision medicine, prognosis factors such as vascular endothelial growth factor (VEGF), serum HER-2 and EGFR, endothelial nitric oxide synthase (eNOS), thrombospondin-1 (THBS-1), circulating endothelial cells (CECs), and gene polymorphism should be considered for patient selection to standardize MCT [18,26,28,32,43].

This meta-analysis has several limitations. First, the significant heterogeneity is a big problem that we cannot bypass. Possible sources may include the differences in study methodologies, treatment history, histopathologic subtypes, and number of participants. We used a random effects model for all analysis in an attempt to minimize this bias. Second, in spite of the fact that we excluded studies with significant missing data, not every study included in this meta-analysis had complete data available. Third, we extracted most of survival data by digitalizing related figures which led to inevitable deviations. Fourth, individual patient data could not be acquired and only two subgroup analyses were performed. Finally, because most of included studies are single-arm trials, the evidence quality was graded to very low, which indicates the present results should be summarized cautiously.

In conclusion, we have comprehensively assessed the use of MCT in MBC treatment by involving 22 phase II or III clinical trials in this meta-analysis. MCT may be a promising therapeutic method for MBC patients, with a favorable tumor response, survival rate, and low toxicity profile. In addition, perhaps combinations of MCT with other conventional anti-cancer therapies did not necessarily improve clinical outcomes. The findings of this meta-analysis show a trend that MCT alone possibly imparts a lower severe AEs rate as compared to the MCT combination schemes. Well-designed RCTs are urgently needed to normalize treatment regimens and to confirm present conclusions.

## Supporting information

S1 Fig Sensitivity analysis on OR (A), CB (B), PFS-6 (C), OS-12(D), OS-24(E) and grade 3/4 AEs (F) rates. (TIF)

S2 Fig Funnel plots for CB (B), PFS-6 (C), OS-12(D), OS-24(E) and grade 3/4 AEs (F) rates. (TIF)

S1 PRISMA Checklist PRISMA 2009 checklist. (DOC)

S1 Table Detailed MCT schedules and registration numbers of the trials included in the meta-analysis. (DOC)

S2 Table GRADE evidence quality assessment: MCT for MBC. (DOC)
