# Supplementary figures and images for: The efficacy and toxicity profile of metronomic chemotherapy for metastatic breast cancer: A meta-analysis
Source: PLoS One. 2017 Mar 15;12(3):e0173693. doi: 10.1371/journal.pone.0173693 (PMC5351982; doi:10.1371/journal.pone.0173693)

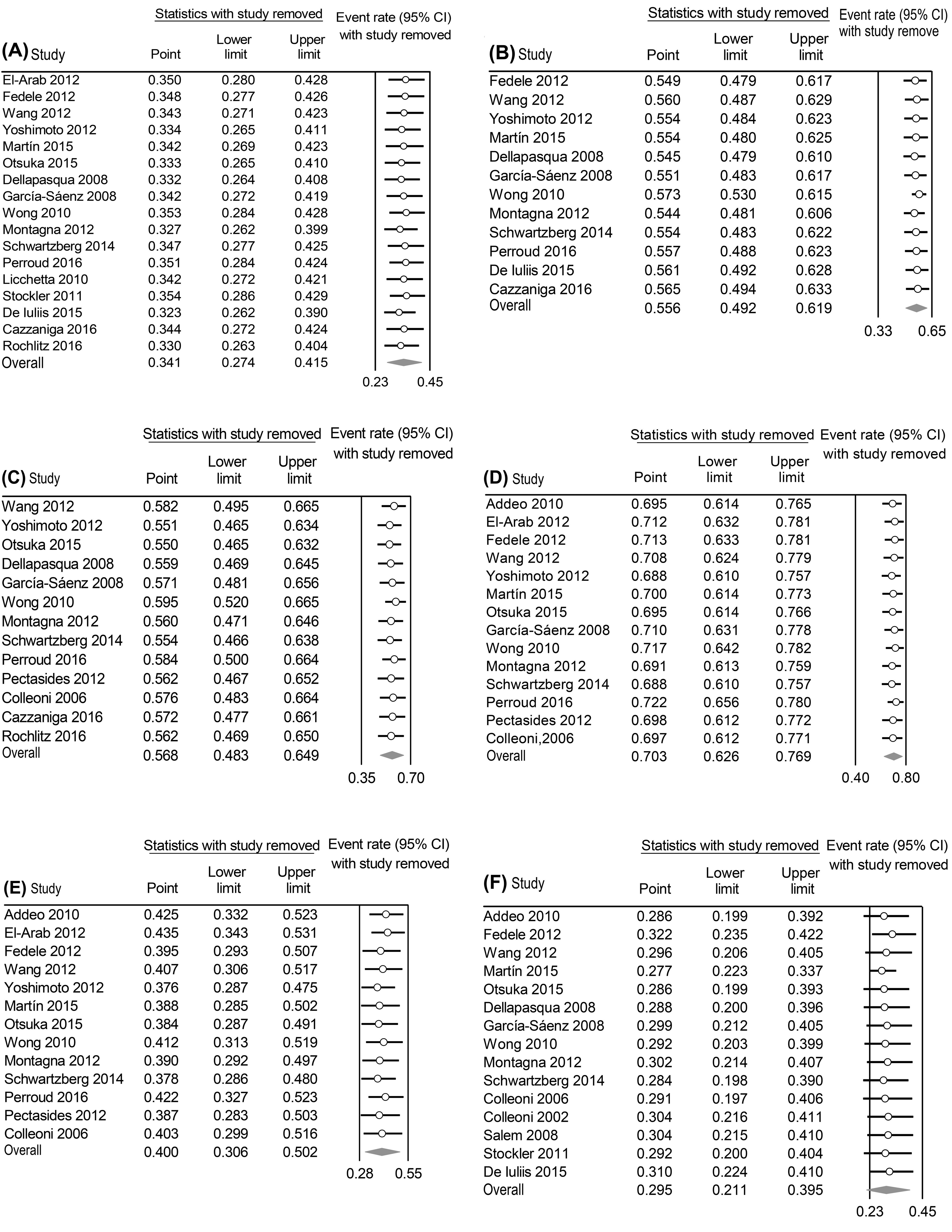

Supplement: S1 Fig — (TIF) [file pone.0173693.s001.tif]

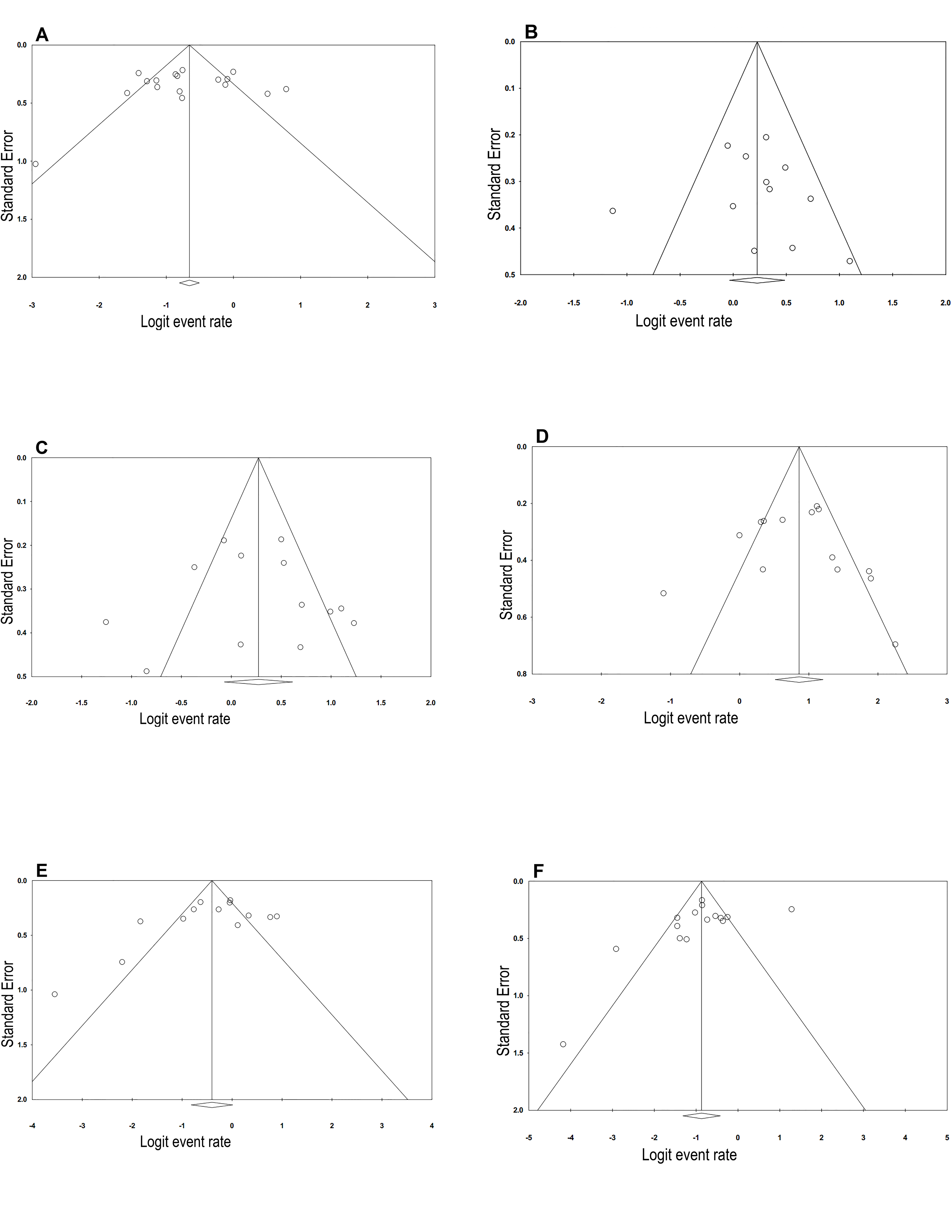

Supplement: S2 Fig — (TIF) [file pone.0173693.s002.tif]
