# Supplementary material for: The efficacy and toxicity profile of metronomic chemotherapy for metastatic breast cancer: A meta-analysis
Source: PLoS One. 2017 Mar 15;12(3):e0173693. doi: 10.1371/journal.pone.0173693 (PMC5351982; doi:10.1371/journal.pone.0173693)
Supplement: S1 Table — (DOC) [file pone.0173693.s004.doc]

| **S1 Table.** Detailed MCT schedules and registration numbers of the trials included in the meta-analysis | | | |
| --- | --- | --- | --- |
| Type | Author，year | Schedule | Registration numberr |
| MCT | Colleoni,2002 | CTX,50 mg/d +MTX 2.5 mg twice daily d1,2 q7 | NA |
|  | Salem,2008 | CTX,50 mg/d +MTX 2.5 mg twice daily d1,2 q7 | NA |
|  | Addeo,2010 | Vinorelbine 70 mg/m^2^ d1,3,5 q14 | NA |
|  | Stockler,2011 | Cap 650mg/m^2^ twice daily | ACTRN12606000379516 |
|  | El-Arab,2012 | CTX 50 mg/d+ Cap 500 mg twice daily | NA |
|  | Fedele,2012 | Cap 1,500 mg/day | NA |
|  | Wang,2012 | CTX 65 mg/m^2^/day d1–14 +Cap 1,000 mg/m^2^ twice daily d1–14 q21 | NCT00589901 |
|  | Yoshimoto,2012 | CTX 33 mg/m^2^/day d1–14+Cap 828 mg/m^2^ twice daily d1–14 q21 | NA |
|  | De Iuliis, 2015 | Vinorelbine 30 mg one day on and one day off | NA |
|  | Martín,2015 | Cap 800mg/m^2^ twice daily | NCT00418028 |
|  | Otsuka,2015 | Irinotecan 60 mg/m^2^ day 1, 8 ,15+TS-1 80 mg/m^2^/day day 3–7, 10–14, 17–21 q28 | KSCOG BC-01 |
|  | Cazzaniga,2016 | Vinorelbine 40 mg thrice daily +Cap 500mg thrice daily | VICTOR-2 |
| Combination | Dellapasqua,2008 | Bevacizumab 10 mg/kg q14+ CTX 50 mg/d+ Cap 500 mg thrice daily | NA |
|  | García-Sáenz,2008 | CTX 50 mg/d+ MTX 1 mg/kg +bevacizumab 10 mg/kg  (trastuzumab in HER2+) q14 | NA |
|  | Wong,2010 | CTX,50 mg/d +MTX 2.5 mg twice daily d1,4 q7 +deltaparin 5,000 UI/d + prednisone 5 mg/d | NA |
|  | Licchetta,2010 | CTX,50 mg/d d1-21 q28 +megestrol acetate 80mg twice daily | NA |
|  | Montagna,2012 | CTX 50 mg/d+Cap 500 mg thrice daily +Erlotinib 100 mg/d+Bevacizumab 15 mg/kg q21 | NA |
|  | Schwartzberg,2014 | Fulvestrant 500 mg d1→250mg d14,29 followed 250mg q28 +Cap 1,500–2,000 mg/d | NCT00534417 |
|  | Perroud,2016 | CTX 50/d+ celecoxib 200 mg twice daily | ANMAT#4596/09 |
|  | Rochlitz,2016 | CTX,50 mg/d +Cap 500mg thrice daily +bevacizumab 10 mg/kg q14 | NCT01131195 |
| Either | Pectasides,2012 | Docetaxel 35mg/m^2^ q7(trastuzumab in HER2+) | HE11/06 |
|  | Colleoni,2006 | Arm A:CTX 50 mg/d +MTX 2.5 mg twice daily d1,4 q7 | NA |
|  |  | Arm B:CTX 50 mg/d +MTX 2.5 mg twice daily d1,4 q7 +thalidomide 200 mg/d |  |
| CTX, cyclophosphamide; MTX, methotrexate; Cap, capecitabine; TS-1, tegafur–gimeracil–oteracil potassium; HER, human epidermal growth factor receptor; NA not available | | | |
|  |  |  |  |
