# Supplementary material for: The efficacy and toxicity profile of metronomic chemotherapy for metastatic breast cancer: A meta-analysis
Source: PLoS One. 2017 Mar 15;12(3):e0173693. doi: 10.1371/journal.pone.0173693 (PMC5351982; doi:10.1371/journal.pone.0173693)
Supplement: S2 Table — (DOC) [file pone.0173693.s005.doc]

| **S2 Table.** GRADE evidence quality assessment: MCT for MBC (randomized trials) | | | | | |
| --- | --- | --- | --- | --- | --- |
| Clinical endpoint (No. of trials) | Bias risk | Inconsistency | Indirectness | Imprecision | Evidence quality |
| OR(n=17) | Very serious risk^b^ | Serious risk^c^ | No serious risk^d^ | No serious risk^e^ | Very low |
| CB(n=12) | Very serious risk^b^ | Serious risk^c^ | No serious risk^d^ | No serious risk^e^ | Very low |
| PFS-6(n=13) | Very serious risk^b^ | Serious risk^c^ | No serious risk^d^ | No serious risk^e^ | Very low |
| OS-12(n=14) | Very serious risk^b^ | Serious risk^c^ | No serious risk^d^ | No serious risk^e^ | Very low |
| OS-24(n=13) | Very serious risk^b^ | Serious risk^c^ | No serious risk^d^ | No serious risk^e^ | Very low |
| Grade 3/4 AEs (n=15) | Very serious risk^b^ | Serious risk^c^ | No serious risk^d^ | No serious risk^e^ | Very low |
| Grade 3/4 AEs^a^ (n=14) | Very serious risk^b^ | Serious risk^c^ | No serious risk^d^ | No serious risk^e^ | Very low |
| ^a^Grade 3/4 AEs after removing a controversial trial | | | | | |
| ^b^Single-arm studies, no internal comparator | | | | | |
| ^c^Obvious heterogeneity | | | | | |
| ^d^Without a comparator, not direct, but this problem is covered under bias risk | | | | | |
| ^e^No events, but owing to lack of comparator this problems is covered under bias risk. | | | | | |
|  |  |  |  |  |  |
